# The Effect of T Regulatory Cell Infiltration on Survival Outcomes in Metastatic Pancreatic Cancer Patients with a Review of Immunobiology, Prognostic Value and Future Therapeutic Options

**DOI:** 10.3390/jcm14238394

**Published:** 2025-11-26

**Authors:** Derya Kıvrak Salim, Canan Sadullahoglu

**Affiliations:** 1 Medical Oncology Department, SBU Antalya Education and Research Hospital, Varlık Mahallesi, Kazım Karabekir Cd., Antalya 07100, Turkey; 2 Pathology Department, SBU Antalya Education and Research Hospital, Varlık Mahallesi, Kazım Karabekir Cd., Antalya 07100, Turkey; canan-rana@hotmail.com

**Keywords:** pancreatic cancer, Tregs, T cell immune response

## Abstract

**Background/Objectives**: Pancreatic ductal adenocarcinoma (PDAC) is one of the most lethal malignancies, with limited therapeutic options despite rapid progress in the immunotherapy era. The balance among CD4^+^ helper T cells (Th), CD8^+^ cytotoxic T cells (Tc), and regulatory T cells (Tregs) is a central determinant of tumor immune dynamics and clinical outcomes. The profound immune suppression in PDAC, driven largely by regulatory T cells (Tregs), remains a major barrier to successful immunotherapy response. Tregs enforce tolerance, shape fibroblasts’ immunosuppressive effect, and reprogram the tumor metabolic niche. This study describes the effect of the relative abundance of effector T cell subtypes and Tregs on survival outcomes in metastatic pancreatic cancer patients and reviews how Tregs and other effector T cell subtypes regulate PDAC immunobiology and influence clinical outcomes. **Methods**: This retrospective study provides immunohistochemical profiling of 62 metastatic PDAC patients, revealing differential prognostic associations among intratumoral and peritumoral subsets of Th, Tc, and Tregs. For each immunostaining, the immune cell infiltrates were evaluated by counting the number of positive cells under the objective of X20 magnification per 0.125 mm^2^. **Results**: While high intratumoral Th (>16.8) and Tc (>19.6) abundances correlated with improved overall survival and progression-free survival, Treg infiltration (both IT and PT) showed no significant prognostic effect. **Conclusions**: The effector Th and Tc are the dominant prognostic T cell subsets in PDAC, whereas Treg abundance alone is an incomplete surrogate of immunosuppression. These findings describe the immunobiological landscape of PDAC.

## 1. Introduction

Pancreatic ductal adenocarcinoma (PDAC) is one of the most fatal malignancies, with an 8% 5-year survival rate. Metastatic disease may have only a 3% 5-year survival rate, which represents a major cause of cancer mortality worldwide [1]. Despite advances in surgical resection, adjuvant chemotherapy, and targeted strategies, the median survival remains less than 12 months for most patients [2]. The poor outcomes of PDAC are attributed to early metastatic spread, genomic complexity, and most notably, a profoundly immunosuppressive tumor microenvironment (TME) [3]. Unlike immunogenic hot cancers such as melanoma or lung carcinoma, PDAC is characterized by a dense desmoplastic stroma that acts as both a physical and biochemical barrier against tumor-infiltrating lymphocytes [4]. Recent studies reveal that the balance among Th, Tc, and Tregs is a central determinant of tumor immune dynamics and clinical outcomes. The emerging evidence indicates that Tc-mediated cytotoxicity remains the strongest correlate of improved survival, whereas Th subsets support cytotoxic function through cytokine signaling, antigen presentation enhancement, and metabolic cooperation. Conversely, Tregs, shaped by hypoxia, lactate, and fibroblast-driven cues, contribute to immune exclusion by suppressing antigen presentation and Tc activation [5,6,7]. Spatially resolved multi-omics have revealed distinct niches in which Th, Tc, and Tregs are differentially enriched, with Tc and activated Th subsets clustering closer to tumor nests, while Tregs often accumulate at stromal–tumor interfaces [8,9]. Within this microenvironment, immune evasion is orchestrated by multiple suppressive immune cell types, including tumor-associated macrophages (TAMs), myeloid-derived suppressor cells (MDSCs), and regulatory T cells (Tregs). Of these, FOXP3^+^ Tregs have emerged as the key conductors of tumor-induced immune tolerance [10]. Tregs, classically defined as CD4^+^CD25^+^FOXP3^+^ lymphocytes, maintain immune homeostasis and prevent autoimmune reactions under physiological conditions [11]. However, in the context of malignancy, Tregs are assigned by tumors to suppress antitumor immunity. They achieve this through immunosuppressive cytokine secretion (IL-10, TGF-β, IL-35), metabolic reprogramming, and engaging inhibitory receptors such as CTLA-4 and PD-1 [12]. These actions collectively silence cytotoxic T lymphocyte (Tc) responses and diminish antigen presentation by dendritic cells. Consequently, Tregs are not merely bystanders but rather key architects of immune exclusion in PDAC [13].

Immunotherapy is an attractive strategy because recent studies have shown that tumor-infiltrating Tc are effective in the microenvironment and are associated with good prognosis, while Treg infiltration mediates immune escape and causes antitumor treatment failure and progression [14,15]. The accumulation of Tregs within the stromal compartment correlates with reduced effector T cell infiltration, resistance to immune checkpoint blockades, and poor overall survival. Additionally, metabolic adaptations allow Tregs to function in different environments, which is important for peripheral immune tolerance that weakens anti-cancer immunity in the tumor microenvironment [16,17,18]. This plasticity explains how Tregs adapt to suppress antitumor immunity even under extreme metabolic stress.

The immune cells infiltrating the tumor and the microenvironment are thought to be critical for cancer outcomes. Predictive and prognostic biomarkers have also been investigated in previous studies to identify subgroups that may benefit from treatments [15,19,20]. The modern therapeutic paradigm, therefore, emphasizes the modulation rather than the ablation of Treg function, seeking to selectively impair their suppressive machinery without disrupting immune homeostasis [21,22]. Recently emerging prognostic models based on T cell-related gene signatures and spatial immune scores are beginning to integrate Th, Tc, and Treg markers to stratify PDAC patients for risk and potential immunotherapy benefits [23]. In this study, our primary aim was to determine the relative abundance of T cell subtypes (CD8^+^ cytotoxic T cells, CD4^+^ helper T cells, and FOXP3^+^ Tregs) and their effect on survival outcomes in metastatic PDC patients.

## 2. Materials and Methods

### 2.1. Study Design and Patients

This study is a cross-sectional, observational, and retrospective clinical trial conducted at Antalya Education and Research Hospital in Turkey. The study protocol was approved by the local ethics committee (protocol no.: 2018-203; approval no.: 8/3; date: 7 March 2019). Informed consent was obtained from all individual participants included in the study.

All patients who were previously diagnosed with metastatic pancreatic cancer and treated in the medical oncology clinic of Antalya Training and Research Hospital between 2013 and 2018 were screened for the study. Inclusion criteria were as follows: age between 18 and 70; histological diagnosis of pancreatic adenocarcinoma; and having adequate tumor tissue samples from the pancreatic tumor area for immune-histochemical analysis. Exclusion criteria were as follows: receiving cancer treatment before tissue sampling; having an autoimmune or inflammatory disease; taking immunomodulators or undergoing immunosuppressive therapy; or having a synchronous second malignancy. The sample size was determined by the availability of eligible archival tissue samples due to the retrospective nature of the study. Sixty-two patients who met the inclusion and exclusion criteria were included in this study.

The first-line treatment protocols were gemcitabine monotherapy or combination therapies (platine with gemcitabine/taxane; nabpaclitaxel–gemcitabine or 5-fluorouracil, leucovorin, irinotecan, and oxaliplatin (FOLFIRINOX)).

### 2.2. Immunohistochemical Staining

All histomorphological data were reviewed from the corresponding hematoxylin and eosin (H&E)-stained slides. The blocks were cut at 4 μm. They were immunostained for CD4 (Leica, CD4/368, 1:100, pre-treatment with Tris buffer at 95° for 20 min; AEC chromogen), CD8 (Leica, CD8/4B11, 1:100, pre-treatment with Tris buffer at 95° for 20 min; AEC chromogen), CD25 (Leica, PA0305, 1:150, pre-treatment with Tris buffer at 95° for 20 min; AEC chromogen), and FOXP3 (Abcam, clone 236A/E7, 1:50, pre-treatment in citrate buffer, 30′, 100 °C). Staining was performed using a Bond Max Autostainer (LEICA Bond III platform) from Leica Microsystem (Wetzlar, Germany). Hematoxylin counterstaining was performed. Tonsil tissue was used as a positive control.

Immunohistochemical monoplex stains (for CD8, CD4, CD25, and FOXP3) were used on serial sections in order to identify Tregs on deparaffinized tissue sections. Firstly, we cut serial sections (ideally 3–4 µm) from the same block. Then, we stained one for CD4, one for CD25, and one for FOXP3. Lastly, we evaluated the Tregs under a microscope as follows:

1. We picked matching anatomical landmarks (tumor glands, ducts, vessels, nerves, fibrosis) to align the three slides.
2. We identified the areas with dense CD4^+^ T cell infiltrates (peritumoral stroma and tumor nests).
3. In those same foci on the FOXP3 slide, we counted nuclear FOXP3^+^ lymphocytes.
4. In the corresponding area on the CD25 slide, we checked that many of those lymphocytes showed clear membranous CD25.

To summarize “the area rich in CD4^+^ and corresponding sections of lymphocytes from the same area had staining for both nuclear FOXP3^+^ and membranous CD25^+^”, we interpreted these as Tregs. Both the intratumoral and peritumoral distribution of T cell subsets were counted.

### 2.3. Immune Scoring

**Figure 1:**
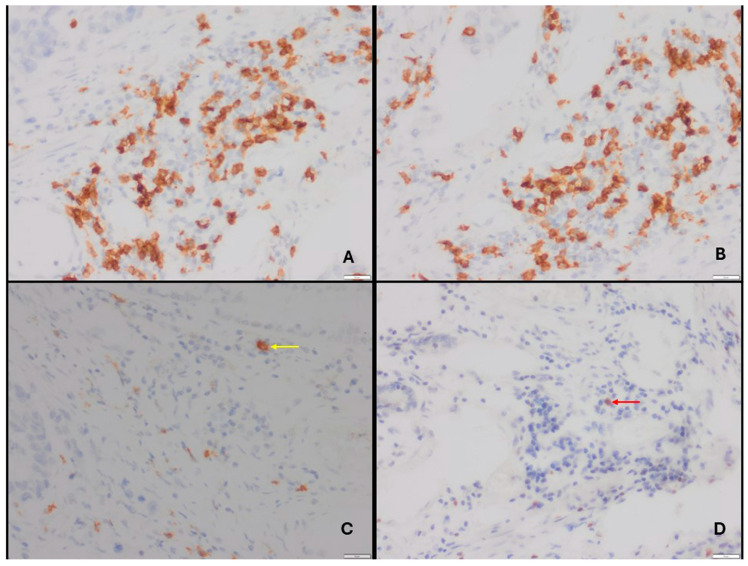
(**A**,**D**) Pancreatic carcinoma. (**A**,**B**) Immunohistochemical demonstration of abundant CD4^+^ and CD8^+^ positive cells in the peritumoral area (DAB, ×400). (**C**,**D**) Immunohistochemical demonstration of fewer CD25^+^ (yellow arrow) and FoxP3^+^ (red arrow) positive cells in the peritumoral area (DAB, ×400).

**Figure 2:**
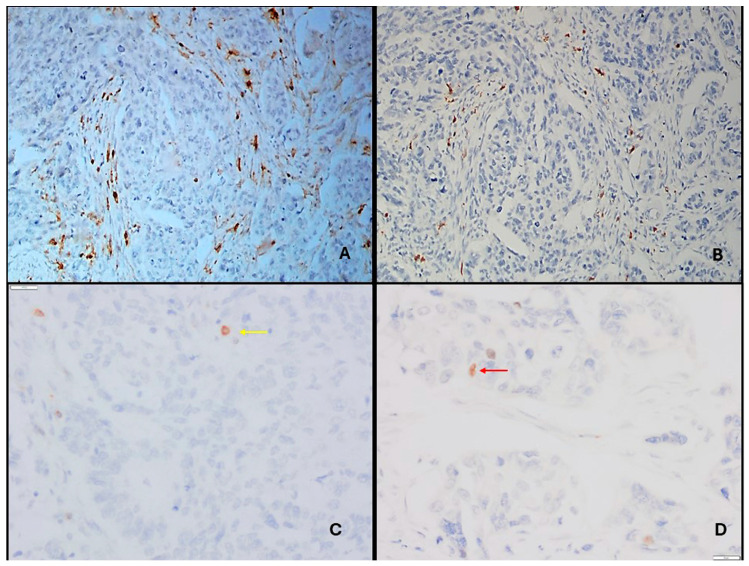
(**A**,**D**) Pancreatic carcinoma. (**A**,**B**) Immunohistochemical demonstration CD4^+^ and CD8^+^ positive cells within the intratumoral area (DAB, ×200). (**C**,**D**) Immunohistochemical demonstration CD25^+^ (yellow arrow) and FoxP3^+^ (red arrow) positive cell within the intratumoral area (DAB, ×400).

The slides were screened under ×10 magnification (Nikon Eclipse Ci-L microscope). Images of the cells were captured under ×20 magnification with a Nikon DS-U3 camera (Nikon Corporation, Shinagawa, Tokyo, Japan). For each immunostaining, the immune cell infiltrates were evaluated by counting the number of positive cells in the intratumoral and peritumoral areas per 0.125 mm^2^. Lymphocytes in the fibrous tissue were counted as peritumoral (stromal), and lymphocytes between at least two tumor cells were counted as intratumoral. The five most intensely stained intratumoral (IT) and peritumoral (PT) areas were counted for each patient (Figure 1 and Figure 2), and mean values were calculated as abundance in order to use in statistical analysis. The ratio of the intratumoral abundance of one type of T cell to peritumoral abundance or the ratio of two different T cell abundances in one area (IT or PT) were defined as relative abundance.

### 2.4. Statistical Analysis

Statistical analysis was performed using IBM SPSS Statistics for Windows, Version 23.0 (IBM Corp., Armonk, NY, USA). Normality assumptions were controlled by the Shapiro–Wilk test. Descriptive analyses were presented using median (min–max) or *n* (%) where appropriate. The abundance of T cell subsets was described as the median of calculated means. The relative proportion of IT T cells (Th or Tc or Tregs) to PT T cells were accepted as a gradient or relative abundance. Median values for relative abundances of T cell subsets of both the IT and PT areas were determined. Using this median value as the cut-off value, patients were dichotomized, and the effects of lower or higher abundances on survival were evaluated.

Categorical data were analyzed by Pearson’s chi-squared or Fisher’s exact test. The differences between the two groups were evaluated with the Mann–Whitney U test for non-normally distributed data. Overall survival (OS) and progression-free survival (PFS) were estimated using the Kaplan–Meier (KM) method in months (mo). The log-rank test was used to compare survival differences. Spearmen’s correlation was used to determine correlation. A univariate Cox proportional hazards regression model was used to identify prognostic factors. A hazard ratio (HR), with corresponding 95% confidence intervals (95% CIs), was reported. A *p*-value of less than 0.05 was considered statistically significant.

## 3. Results

### 3.1. Clinical and Immunobiological Findings

#### 3.1.1. Patient Demographics and Cellular Distribution

The baseline characteristics of 62 patients are outlined in Table 1.

Peritumoral Th and Tc cell densities were higher than IT densities, whereas Treg densities were comparable in number in both areas (Table 1).

In total, 36 (58.1%) patients had liver metastases, 27 (43.5%) had lung metastases, 3 (4.83%) had bone metastases, 48 (77.4%) had lymph node metastases, and 36 (58.1%) had omental metastases.

While 20 (32.3%) patients received gemcitabine monotherapy, 42 (67.7%) patients received standard combination chemotherapy.

The abundance (density) and relative abundance of T cell subsets are described in Table 1.

#### 3.1.2. Survival and Risk Factors Affecting Survival

Younger age resulted in better OS (17 mo (13.119–20.881) vs. 8 mo (5.585–10.415) *p* < 0.001) and better PFS (9 mo (3.923–14.077) vs. 5 mo (3.303–6.697) *p* = 0.003).

A good performance score (0–1 vs. 2) showed improved survival (*p* = 0.031, *p* = 0.015 for PFS and OS, respectively).

Metastatic area differences did not affect OS or PFS. The interrelationship between clinical and tumoral characteristics and survival is presented in Table 2.

There were no differences in IT and PT Tc infiltration according to tumor size ((OR 0.77; 95% CI 0.3–2.1; *p*: 0.6), (OR 0.45; 95% CI 0.16–1.3; *p*: 0.12)), lymphovascular invasion ((OR 0.6; 95% CI 0.2–2.3; *p*: 0.48), (OR 0.4; 95% CI 0.12–1.7; *p*: 0.22)), perineural invasion ((OR 0.3; 95% CI 0.03–3.1; *p*: 0.3), (OR 0.0; *p*: 0.99)), and grade ((OR 2.2; 95% CI 0.4–13.2; *p*: 0.37), (OR 0.45; 95% CI 0.08–2.6; *p*: 0.37)).

Moreover, there were not any differences in IT and PT Treg infiltration according to tumor size ((OR 1.9; 95% CI 0.69–5.25; *p*: 0.21), (OR 1.69; 95% CI 0.61–4.6; *p*: 0.3)), lymphovascular invasion ((OR 0.89; 95% CI 0.25–3.1; *p*: 0.85), (OR 1.46; 95% CI 0.41–5.2; *p*: 0.56)), perineural invasion ((OR 0.0; *p*: 0.99), (OR 0.0; *p*: 0.99)), and grade ((OR 0.52; 95% CI 0.09–3.1; *p*: 0.47), (OR 0.48; 95% CI 0.08–2.9; *p*: 0.42)).

Intratumoral Th abundance > 16.8 is associated with improved OS (*p* < 0.001). Intratumoral Tc abundance > 19.6 is also associated with better OS (*p* = 0.018).

Peritumoral Tc abundance did not affect OS and PFS (*p* = 0.99, *p* = 0.16, respectively), whereas Th abundance > 41.8 had improved OS and PFS (*p* = 0.003, *p* = 0.011, respectively) (Table 3).

Intratumoral or peritumoral Treg abundances did not influence OS (*p* = 0.179, *p* = 0.330, respectively) or PFS (*p* = 0.321, *p* = 0.734, respectively).

**Figure 3:**
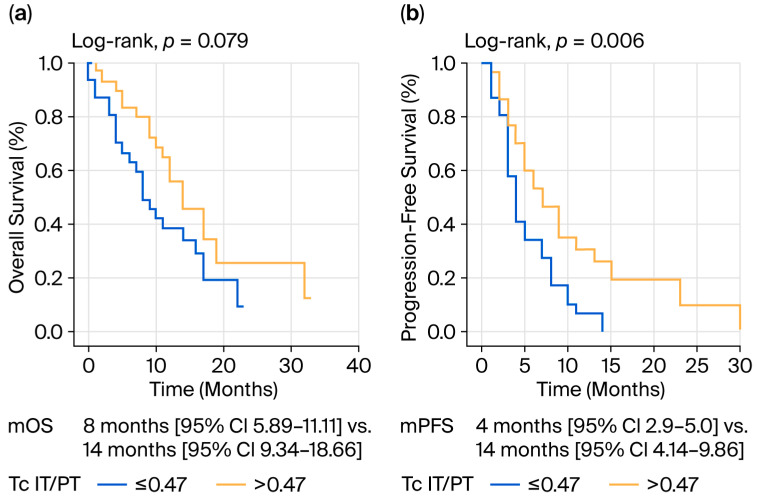
Tc relative abundance and survival: (**a**) Tc relative abundance and OS; (**b**) Tc relative abundance and PFS.

Multivariate analysis showed that IT Th abundance < 16.8 had increased mortality risk (HR 3.27; 95% CI, 1.17 to 9.15; *p* = 0.024) and Tc relative abundance (IT/PT) < 0.47 had increased progression risk (HR 2.43; 95% CI, 1.3 to 4.55; *p* = 0.005) (Table 3 and Table 4, Figure 3).

**Figure 4:**
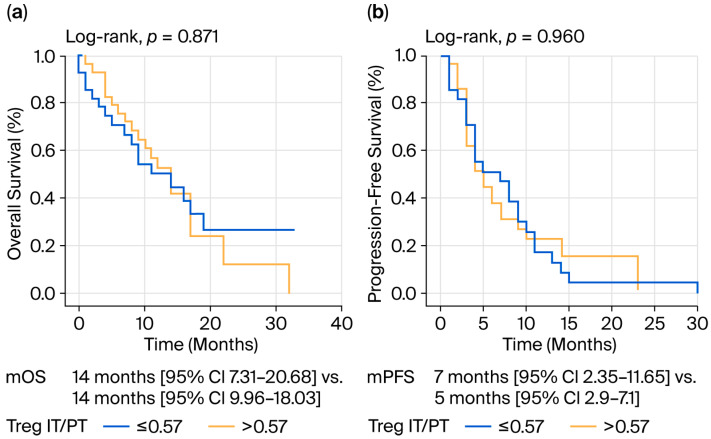
Treg relative abundance and survival: (**a**) Treg relative abundance and OS; (**b**) Treg relative abundance and PFS.

The relative abundance of Tregs (IT/PT) did not affect survival in any way (Table 3 and Figure 4).

There were moderate correlations in Th (r = 0.653, *p* < 0.001) and Tc (r = 0.662, *p* < 0.001) cells and a strong correlation in Tregs (r = 0.747, *p* < 0.001) between IT and PT areas. In addition, there was a weak correlation between Tc and Treg abundance in both IT (r = 0.253, *p* = 0.047) and PT (r = 0.262, *p* = 0.040) areas.

First-line combination chemotherapy treatment was associated with better survival, which was not independently prognostic. The ability to undergo second-line chemotherapy was also found to be a positive prognostic factor (Table 3 and Table 4).

## 4. Discussion

Over the last few years, major advancements in single-cell sequencing and spatial transcriptomics have substantially deepened our understanding of T cell biology in pancreatic ductal adenocarcinoma (PDAC).

### 4.1. Th and Tc Cell Infiltration: Alignment with Existing Evidence

Consistent with the literature, the current study demonstrates that the enhanced infiltration of effector Th and Tc cells within tumor nests predicts better survival. Prior single-cell and transcriptomic analyses have confirmed that PDAC tumors containing activated Tc and IFN-γ-producing Th subsets exhibit more favorable immune profiles and occasionally respond to immunotherapeutic combinations [17,24,25]. The significant prognostic association of high IT Tc density (OS: 14 vs. 8 months; PFS: 7 vs. 4 months, *p* = 0.006) supports translational data, suggesting that the spatial proximity of cytotoxic lymphocytes to tumor glands is a determinant of effective immune surveillance [3]. Mechanistically, Tc cells require sustained metabolic fitness, and their intratumoral persistence is supported by multiple cytokine pathways. Recent studies have only shown that the co-infiltration of pancreatic tumors with Th and Tc is independently prognostic from OS and PFS. Tumor infiltration by Th or Tc alone was not associated with longer survival [14,24]. These discordances could be due to different methodological approaches used to identify lymphocyte subpopulations or histological subtype differences, which have previously been described by Kim et al. [26]. The current findings align with preclinical studies showing that stromal CXCL12 blockade or CD40 activation or nanomedicine treatment induce and restore both Th and Tc infiltration and improve PDAC outcomes [22,27,28,29,30]. Importantly, the correlation between Th and Tc densities in both IT and PT regions, as observed in our study, emphasizes that coordinated helper–cytotoxic interactions remain central to anti-tumor immunity.

### 4.2. Non-Prognostic Role of Tregs: Discrepancy and Interpretation

Contrary to many transcriptomic- and flow cytometry-based reports linking Treg enrichment to poor prognosis in PDAC [19,31,32,33,34], this study found that Tregs were not independently associated with OS or PFS (*p* = 0.871 and 0.960, respectively). Several factors may explain this apparent discrepancy. First, immunohistochemistry (IHC) primarily quantifies Treg density rather than function. Recent multi-omics data indicate that not all FOXP3^+^ cells in PDAC are equally suppressive; subsets such as Helios^−^ or ICOS^+^ Tregs exhibit reduced inhibitory capacity or even tissue repair phenotypes [33,34]. Thus, phenotypic heterogeneity within the FOXP3^+^ population could obscure prognostic associations when only marker expression is evaluated. Second, the spatial compartmentalization of Tregs is of significance. Spatial transcriptomic and multiplex imaging analyses have revealed that Tregs accumulate at tumor–stroma interfaces, where they interact with cancer-associated fibroblasts (CAFs) and myeloid cells [35,36]. These interactions promote immune exclusion without necessarily influencing patient survival directly. The present study’s finding that Treg abundance was similar in IT and PT regions supports the concept of stromal sequestration, where Tregs act more as “barrier architects” than as direct effectors of prognosis. Third, chemotherapy exposure may modulate Treg function. Most patients in this cohort received gemcitabine-based or combination regimens that transiently deplete proliferative Tregs [25,37,38]. Therefore, baseline Treg infiltration might lose prognostic weight in treated metastatic settings.

### 4.3. Integrating Translational Mechanisms with Clinical Observations

The convergence between high Th/Tc infiltration and favorable outcomes highlights the critical role of effector T cell engagement in PDAC biology. This echoes the findings from preclinical models showing that tumor microenvironment therapeutic reprogramming via CXCR4 blockade, CD40 agonists, or TGF-β inhibition enhances Tc recruitment and restores anti-tumor activity [27,28,29,39,40,41]. In contrast, Treg-targeting strategies such as anti-CD25 depletion or CD40/CXCR4/TGF-β inhibition yield variable outcomes, underscoring the need for selective modulation rather than global suppression. The current IHC data, thus, refine translational paradigms: Treg quantity alone is insufficient as a prognostic or therapeutic biomarker; instead, qualitative metrics—such as metabolic signatures, cytokine production, and spatial context—should be prioritized for investigation. This is in line with recent integrative analyses demonstrating that Treg function is governed by the metabolic microenvironment and cytokine gradients rather than absolute abundance [36,42].

### 4.4. Biological and Methodological Considerations

The present clinical results further support the “immune–spatial equilibrium” model described in recent reviews. PDAC outcomes appear determined not merely by immune cell counts but by their spatial arrangement and interaction dynamics [36,37,38,39,40,41,42]. The observation that peritumoral Tc abundance lacked prognostic impact mirrors prior findings that stromal Tc infiltration remains functionally excluded by CAF-derived barriers [35,36]. In contrast, the protective effect of intratumoral Th and Tc enrichment underscores the therapeutic potential of overcoming physical and biochemical exclusion zones. From a methodological perspective, this study’s dual assessment of IT and PT compartments provides valuable granularity. Future work may combine IHC with multiplex spatial assays or RNA in situ hybridization to define transcriptionally distinct Treg subsets and effector niches.

### 4.5. Clinical and Translational Implications

The alignment between improved survival and high IT effector T cell infiltration supports integrating immune cell spatial quantification into PDAC prognostication algorithms. Automated digital pathology systems could facilitate standardized immune scoring, complementing molecular assays to identify patients most likely to benefit from immunotherapy. Furthermore, the absence of a Treg–survival correlation challenges the assumption that Treg depletion uniformly benefits PDAC patients. Instead, selective reprogramming through CCR4 or CXCR4 antagonists, adenosine pathway inhibitors, or bispecific antibodies targeting PDL1 and TGF may yield better therapeutic windows [27,28,29,39,40,41].

In summary, Tregs represent a dynamic and adaptable cell population within PDAC that coordinates immune evasion, stromal reorganization, and metabolic adaptation. Understanding the molecular programs that govern this plasticity is critical for the rational design of immunotherapeutic strategies that selectively reprogram rather than eradicate Tregs. Thus, the Treg–stromal–myeloid axis stands as one of the most critical barriers to immunotherapy success in pancreatic cancer and remains a central target for ongoing translational research.

### 4.6. Strength of Positive Prognostic Role of Intratumoral Effector T Cells

One of the main strengths of this study is the clear demonstration of high IT Th and Tc cell abundance being strongly associated with prolonged survival. Patients with elevated IT Th (>16.8) and Tc (>19.6) infiltrates showed significantly longer OS and PFS, consistent with previous reports demonstrating that effector T cell-rich tumors exhibit more favorable immunologic activity and clinical outcomes [3,17,24]. These findings highlight that effector T cell infiltration—not Treg abundance—is the dominant immune correlate of improved prognosis in metastatic PDAC.

This concept aligns with the broader literature demonstrating that pancreatic cancer, traditionally viewed as an “immune-cold” tumor, contains rare but clinically meaningful pockets of immune activation characterized by Tc infiltration and IFN-γ-producing Th subsets [17,24]. The positive correlation observed between Th and Tc abundance across IT and PT regions in our study further supports the coordinated nature of anti-tumor immune responses. Such interdependent concept means that Th–Tc crosstalk is essential for effective cytotoxic responses, functioning through antigen presentation enhancement and T cell metabolic support within the suppressive PDAC microenvironment [24,26]. Wang et al. demonstrated that spatial proximity and coordinated clustering of Th and Tc within tumor nests correlate strongly with improved survival in PDAC. Their work underscored that abundance measurements may underestimate the functional relevance of effector T cell activity; rather, co-infiltration and spatial cooperation are essential determinants of effective anti-tumor responses [43]. Our study’s findings closely mirror this principle. The moderate-to-strong correlations between Th and Tc densities in both IT and PT compartments (r ≈ 0.65) indicate synchronized immune activation, and the high IT abundance of both subsets independently predicts superior OS and PFS. These patterns reinforce the concept that Th–Tc immune circuits are critical for mounting productive anti-tumor immunity, especially in desmoplastic tumors such as PDAC.

### 4.7. Future Therapeutic Directions

The present study demonstrates that IT effector T cell infiltration is a superior prognostic marker compared to Treg abundance in metastatic PDAC. These results support the development of therapeutic strategies that involve remodeling the TME to enhance effector T cell penetration. Given the strong prognostic value of IT Th and Tc abundance in this study, emerging therapeutic models should prioritize the following:

a. Enhancing T cell recruitment into tumor nests (e.g., CXCR4/CXCL12 inhibitors, CD40 agonists).
b. Reprogramming suppressive stromal and myeloid networks (CAF-dominated stroma) that limit effector T cell access.
c. Selective, not global, Treg modulation, such as through CCR4 or CXCR4 antagonism, adenosine pathway blockade, or bispecific antibodies (e.g., PD-L1/TGF-β inhibitors).
d. Developing spatially informed biomarkers based on digital pathology, multiplex IHC, and single-cell technologies.

To validate the prognostic and therapeutic relevance of spatial immune metrics, prospective, multi-institutional clinical studies are needed. Such studies should integrate spatial profiling, single-cell transcriptomics, and functional Treg characterization to clarify which immune cell configurations most accurately predict treatment response and survival.

### 4.8. Limitations

A major limitation of this paper is the very small sample size and lack of adequate controls. Also, the kinetics and migration of FOXP3 Tregs infiltrating into PDC and their microenvironment may differ with methodological analysis, such as immunophenotyping with a flow cytometer or immunohistochemical staining. Treatment differences may also be confounding factors for survival analysis.

While double-blind evaluation by two separate pathologists would have been a more reliable method for evaluating immune scores and immune subpopulations, unfortunately, our study was limited in this regard.

True triple IHC (CD4/CD25/FOXP3 on the same cell) is more precise. With monoplex chromogenic IHC, our identification was inferred (serial sections + morphology). This was another limitation of our study.

## 5. Conclusions

Our findings fit well with the emerging view that effectors Th and Tc are the dominant prognostic T cell subsets in PDAC, whereas Treg abundance alone is an incomplete surrogate for immunosuppression.

The combined interpretation of this clinical cohort and the broader literature suggests that future PDAC trials should stratify patients not only by Treg frequency but also by their localization and functional phenotype. The integration of single-cell, spatial, and computational profiling into clinical studies will be essential to distinguish suppressive from reparative Treg populations. These insights may inform the biomarker-driven application of combination immunotherapy and stromal modulation strategies.

## Figures and Tables

**Table 1:** Patient demographics and T cell infiltration patterns.

	*n* = 62
Age, median (min–max)	66 (47–86)
Gender (male/female)	37(59.7)/25(40.3)
ECOG PS, median (min–max)	1 (0–3)
<2	43 (69.4)
≥2	19 (30.6)
Tumor size, median (min–max)	3 (1–9.5)
LVI, *n* (%)	49 (80.3)
PNI, *n* (%)	57 (93.4)
Grade, *n* (%)	
1	1 (1.6)
2	54 (88.6)
3	6 (9.8)
PT, median (min–max)	
Th PT	41.8 (0–181)
Tc PT	47.4 (0–122)
Treg PT	2.9 (0–62.8)
IT, median (min–max)	
Th IT	16.8 (0–216)
Tc IT	19.6 (0–100)
Treg IT	2.2 (0–27)
Relative abundance (density gradient)	
Th IT/PT	0.39 (0.04–2.3)
Tc IT/PT	0.47 (0.04–1.76)
Treg IT/PT	0.57 (0–14)

LVI: lymphovascular invasion; PNI: perineural invasion; PT: peritumoral infiltration; IT: intratumoral infiltration; Th: T helper; Tc: T cytotoxic; Treg: regulatory T cell.

**Table 2:** Univariate analysis of risk factors affecting survival.

	OS		PFS	
	Median Survival, Months (95% CI)	*p*	Median Survival, Months (95% CI)	*p*
Gender				
Female	16 (10.839–21.161)	0.160	7 (4.681–9.319)	0.326
Male	9 (5.664–12.336)		5 (3.082–6.918)	
Tumor size				
≤3	16 (12.326–19.674)	0.124	5 (2.619–7.381)	0.174
>3	9 (7.322–10.678)		4 (0.215–7.785)	
LVI				
No	14 (10.124–17.876)	0.190	6 (1.371–10.629)	0.351
Yes	10 (7.235–12.765)		5 (3.028–6.972)	
PNI				
No	2 (0–12.78)	0.248	5 (0–10.988)	0.911
Yes	12 (8.266–15.734)		5 (2.822–7.178)	
Grade				
1–2	12 (7.744–16.256)	0.430	5 (3.081–6.919)	0.118
3	8 (0–17.602)		4 (1.737–6.263)	
Liver metastases				
No	12 (6.271–17.729)	0.803	6 (3.084–8.916)	0.265
Yes	11 (6.038–15.962)		4 (2.576–5.424)	
Lung metastases				
No	9 (5.758–12.242)	0.472	6 (2.702–9.298)	0.364
Yes	14 (8.978–19.022)		5 (3.749–6.251)	
Bone metastases				
No	12 (8.663–15.337)	0.395	5 (3.302–6.698)	0.898
Yes	9 (0–20.202)		6 (NA)	
Lymph node metastases				
No	14 (11.69–16.31)	0.334	8 (6.952–9.048)	0.221
Yes	10 (6.59–13.41)		4 (2.701–5.299)	
Omental metastases				
No	14 (8.413–19.587)	0.262	6 (4.195–7.805)	0.506
Yes	10 (6.056–13.944)		5 (3.864–6.136)	
First-line chemotherapy type				
Gemcitabine mono	8 (4.329–11.671)	0.001	3 (1.973–4.027)	0.007
Combination chemo	16 (12.174–19.826)		7 (4.009–9.991)	
Second-line chemotherapy				
No	8 (5.27–10.73)	0.004	5 (3.292–6.708)	0.722
Yes	17 (15.805–18.195)		6 (2.636–9.364)	

NA: not applicable; LVI: lymphovascular invasion; PNI: perineural invasion.

**Table 3:** Univariate analysis of immune cell factors affecting survival.

	OS		PFS	
	Median Survival, Months (95% CI)	*p*	Median Survival, Months (95% CI)	*p*
Th IT *				
≤16.8	8 (5.372–10.628)	<0.001	4 (2.976–5.024)	0.001
>16.8	17 (12.525–21.475)		9 (5.946–12.054)	
Th PT *				
≤41.8	9 (6.465–11.535)	0.003	5 (3.53–6.47)	0.011
>41.8	17 (16.045–17.955)		8 (3.766–12.234)	
Tc IT *				
≤19.6	9 (6.489–11.511)	0.018	4 (2.482–5.518)	0.066
>19.6	16 (11.934–20.066)		7 (4.915–9.085)	
Tc PT *				
≤47.4	10 (6.067–13.933)	0.099	5 (3.508–6.492)	0.160
>47.4	16 (11.445–20.555)		6 (2.833–9.167)	
Treg IT *				
≤2.2	10 (7.234–12.766)	0.179	5 (2.836–7.164)	0.321
>2.2	14 (10.392–17.608)		5 (1.41–8.59)	
Treg PT *				
≤2.9	11 (8.058–13.942)	0.330	5 (2.871–7.129)	0.734
>2.9	14 (6.86–21.14)		5 (2.377–7.623)	
Th IT/PT **				
≤0.39	14 (7.363–20.637)	0.531	5 (3.566–6.434)	0.673
>0.39	12 (9.03–14.97)		6 (3.705–8.295)	
Tc IT/PT **				
≤0.47	8 (4.89–11.11)	0.079	4 (2.971–5.029)	0.006
>0.47	14 (9.341–18.659)		7 (4.144–9.856)	
Treg IT/PT **				
≤0.57	14 (7.318–20.682)	0.871	7 (2.35–11.65)	0.960
>0.57	14 (9.961–18.039)		5 (2.9–7.1)	
Treg/Th IT **				
≤0.13	16 (12.001–19.999)	0.059	6 (3.316–8.684)	0.111
>0.13	9 (4.396–13.604)		4 (2.759–5.241)	
Treg/Th PT **				
≤0.06	14 (5.912–22.088)	0.874	6 (3.705–8.295)	0.594
>0.06	11 (6.416–15.584)		4 (2.326–5.674)	
Treg/Tc IT **				
≤0.11	12 (7.087–16.913)	0.681	6 (3.853–8.147)	0.159
>0.11	11 (5.609–16.391)		4 (3.112–4.888)	
Treg/Tc PT **				
≤0.06	12 (8.175–15.825)	0.565	5 (3.461–6.539)	0.450
>0.06	14 (8.881–19.119)		7 (2.996–11.004)	

PT: peritumoral infiltration; IT: intratumoral infiltration; Th: T helper; Tc: T cytotoxic; Treg: regulatory T cell. * abundance; ** relative abundance.

**Table 4:** Multivariate analysis of risk factors affecting survival.

	OS		PFS	
Variables	HR (95% CI)	*p*	HR (95% CI)	*p*
Age				
≤66	Reference	-	Reference	-
>66	1.873 (0.826–4.25)	0.133	1.336 (0.648–2.754)	0.432
ECOG PS				
<2	Reference	-	Reference	-
≥2	0.81 (0.35–1.875)	0.623	2.03 (0.995–4.14)	0.052
First-line chemotherapy type				
Gemcitabine mono	1.534 (0.707–3.325)	0.279	1.316 (0.667–2.597)	0.429
Combination chemo	Reference	-	Reference	-
Second-line chemotherapy				
No	2.434 (1.073–5.518)	0.033		
Yes	Reference	-		
Th IT *				
≤16.8	3.275 (1.171–9.153)	0.024	0.891 (0.32–2.482)	0.826
>16.8	Reference	-	Reference	
Th PT *				
≤41.8			1.956 (0.731–5.229)	0.181
>41.8			Reference	
Tc IT *				
≤19.6	0.795 (0.334–1.889)	0.603		
>19.6	Reference	-		
Tc IT/PT **				
≤0.47			2.437 (1.303–4.556)	0.005
>0.47			Reference	

PT: peritumoral infiltration; IT: intratumoral infiltration; Th: T helper; Tc: T cytotoxic; * abundance; ** relative abundance.

## Data Availability

Additional data for the eligible studies are available upon request from the corresponding author at deryakivrak@gmail.com.

## Abbreviations

The following abbreviations are used in this manuscript:

CAFs	cancer-associated fibroblasts
CIs	confidence intervals
CTL	cytotoxic T lymphocyte
FOXP3	forkhead box P3
H&E	hematoxylin and eosin
HR	hazard ratio
IHC	immunohistochemistry
IT	intratumoral
MDSCs	myeloid-derived suppressor cells
mo	months
OR	odds ratio
OS	overall survival
PFS	progression-free survival
PT	peritumoral
PDAC	pancreatic ductal adenocarcinoma
TAMs	tumor-associated macrophages
Tc	cytotoxic T cells
TGF-β	transforming growth factor beta
Th	T helper cells
TME	tumor microenvironment
Tregs	regulatory T cells
